# Integrated Advanced Echocardiographic and Biomarker Approach for the Early Detection of Atrial Cardiomyopathy in Middle-Aged Patients With Atrial Fibrillation

**DOI:** 10.7759/cureus.88642

**Published:** 2025-07-24

**Authors:** Angelina Borizanova, Elena Kinova, Veselina Koleva, Otilia Tica, Petar R Kalaydzhiev, Assen Goudev

**Affiliations:** 1 Emergency Medicine, Medical University - Sofia, Sofia, BGR; 2 Clinical Laboratory, Acibadem City Clinic Tokuda Hospital, Sofia, BGR; 3 Cardiovascular Sciences, University of Birmingham, Birmingham, GBR

**Keywords:** atrial cardiomyopathy, atrial fibrillation, left atrial reservoir strain, left atrial volume index, obesity

## Abstract

Background

Atrial fibrillation (AF) and atrial cardiomyopathy (AtCM) share overlapping pathophysiological processes, with abnormalities in atrial structure and function being key contributors to disease progression.

Aim

This study aimed to assess echocardiographic parameters and circulating biomarkers in middle-aged AF patients without previously known overt heart disease, to explore early markers of atrial dysfunction and AtCM.

Methods

Prospective, consecutive patients (n = 970) who had been admitted for symptomatic AF to our hospital from January 2016 to January 2018 were screened for participation in the study. A total of 70 patients met the inclusion criteria: stable sinus rhythm, age between 40 and 60 years, and structurally normal hearts assessed by conventional two-dimensional echocardiography (2DE). They were separated into two groups: new-onset AF (n = 33) and recurrent episodes of paroxysmal AF (n = 37). Thirty age-matched healthy subjects were enrolled in the control group. All patients underwent 2DE assessment with volumetric and speckle-tracking analyses. Galectin-3, high-sensitivity troponin I (hsTnI), and high-sensitivity C-reactive protein (hsCRP) were measured.

Results

Significant structural and functional impairments were observed in both atria among AF patients, with the left atrium (LA) showing more pronounced abnormalities. Key parameters, such as LA reservoir and contractile strain, as well as electromechanical delay (EMD), were notably reduced. Individuals with paroxysmal AF exhibited elevated galectin-3 and hsTnI concentrations compared to both new-onset AF patients and controls, indicating more advanced fibrosis and myocardial stress. Furthermore, LA stiffness index and strain measures correlated strongly with galectin-3 levels.

Conclusion

In middle-aged patients without overt heart disease, advanced echocardiographic parameters (particularly strain and EMD), combined with biomarkers such as galectin-3 and hsTnI, represent sensitive markers for early atrial dysfunction and AtCM. The integration of these diagnostic tools may improve early detection, facilitate risk assessment, and support timely therapeutic interventions to prevent the progression of AF.

## Introduction

The concept of atrial cardiomyopathy (AtCM), first introduced in 1972, remained largely overlooked in clinical practice until it was formally defined as a distinct entity in a 2016 expert consensus statement [1]. Since that time, research activity around AtCM has grown substantially, culminating in recent updates that have deepened insights into its pathophysiology and clinical relevance [2].

AtCM includes a diverse range of abnormalities affecting the atrial myocardium, spanning changes in structure, architecture, mechanical function, and electrical conduction. These alterations can result in clinical outcomes such as atrial arrhythmias, increased thromboembolic events, functional impairment of atrial valve competence, and, ultimately, atrial failure [3-5]. It is important to recognize that AF may serve as an early indicator of AtCM, even in individuals without prior diagnosed heart conditions [6].

Currently, AtCM is conceptualized as a progressive disease with multiple stages, for which a classification framework has been suggested by experts [2]. However, further clinical validation of this staging system is needed, as gaps persist in our understanding of disease mechanisms and phenotype diversity. Challenges in distinguishing distinct AtCM phenotypes largely stem from limited empirical data [4].

This study aimed to: (1) characterize changes in atrial morphology and function using advanced echocardiographic techniques in middle-aged patients with AF without previously diagnosed overt heart disease or structural abnormalities; (2) assess the clinical impact of impaired atrial function; and (3) explore correlations between echocardiographic findings and laboratory biomarkers to improve risk stratification.

This article has been previously published as a preprint on Research Square and can be accessed via the following DOI (https://doi.org/10.21203/rs.3.rs-6751760/v1).

## Materials and methods

A prospective study was carried out involving consecutive patients (n = 970) presenting with symptomatic atrial fibrillation (AF), who were admitted to the Cardiology Department of the University Hospital "Tsaritsa Yoanna - ISUL", Sofia, Bulgaria, for restoration of sinus rhythm between January 2016 and December 2018. The diagnosis and classification of AF followed established clinical guidelines [6].

Following the application of strict inclusion criteria - including maintenance of stable sinus rhythm for at least three weeks post-restoration, age between 40 and 60 years, absence of previously diagnosed overt heart disease with structural or functional impairment, preserved left ventricular ejection fraction (LVEF) and global longitudinal strain (GLS), and normal diastolic parameters - 70 patients met the eligibility requirements.

**Figure 1:**
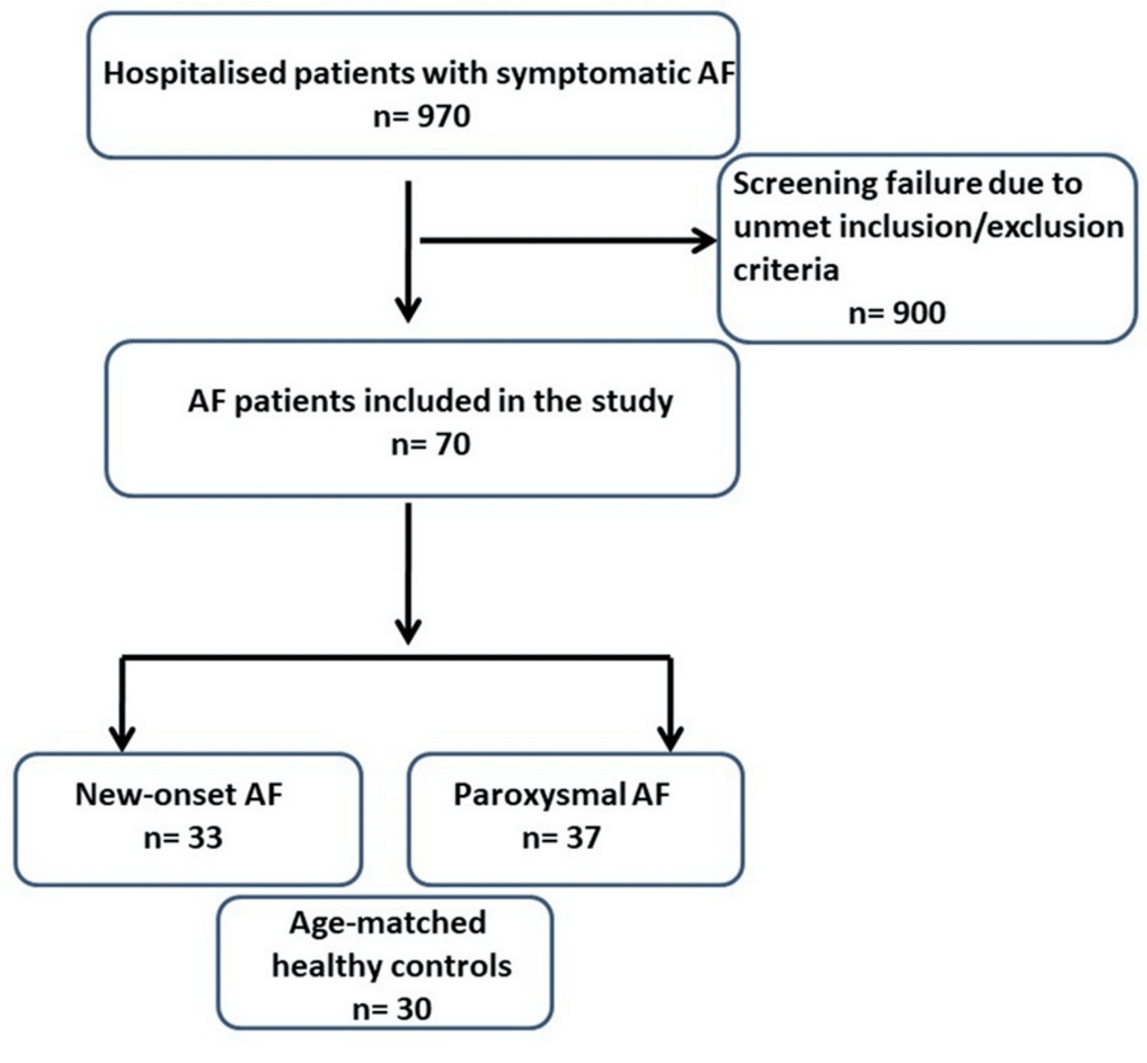
Study flowchart illustrating the total study AF, atrial fibrillation

Patients were excluded if they were older than 60 years or had any of the following: coronary artery disease, valvular or congenital heart defects, persistent arrhythmias other than AF, cardiomyopathies, left ventricular (LV) systolic dysfunction (LVEF <50%), pericarditis, myocarditis, heart failure, chronic obstructive pulmonary disease, diabetes mellitus, thyroid disorders, pacemaker presence, postoperative AF, sleep apnea, anemia, malignancies, alcohol abuse, chronic kidney or liver disease, prior ablation interventions, history of stroke or transient ischemic attack (TIA), or suboptimal echocardiographic image quality (Figure 1).

A control group of 30 age-matched individuals without any previously diagnosed overt heart disease or structural abnormalities was recruited. These controls underwent comprehensive evaluation, including physical examination, laboratory testing, electrocardiography, and standard echocardiography, all yielding normal results. None of the controls had a history of AF, other arrhythmias, hypertension, diabetes, or any cardiovascular or systemic disorders.

Patients with AF identified in the study cohort were prospectively monitored over a five-year period to evaluate the progression of AF to persistent or permanent forms, along with associated clinical outcomes and treatment strategies.

The study protocol was approved by the Ethics Committee of the University Hospital "Tsaritsa Yoanna - ISUL" (approval no. 2145), and all patients provided written informed consent in accordance with the Declaration of Helsinki. All clinical evaluations were conducted after maintaining stable sinus rhythm for at least three weeks.

Laboratory assessment

Venous blood was drawn to analyze a standard biochemical panel, as well as high-sensitivity troponin I (hsTnI), high-sensitivity C-reactive protein (hsCRP), and the fibrotic marker galectin-3. Galectin-3 concentrations were measured using a two-step chemiluminescent microparticle immunoassay, which has a detection limit of 1.0 ng/mL and a linear range from 4.0 to 114 ng/mL. Assay accuracy and precision were ensured by testing control samples at concentrations of 9.1, 20.5, and 74.1 ng/mL. The results were blinded and obtained at the end of the study. Blinding was applied during the biomarker analysis and echocardiographic assessment, so the investigators were unaware of the participants' group allocation.

Echocardiography

Ultrasound examinations were conducted using the Philips EPIQ7 system (Philips Healthcare, Amsterdam, the Netherlands), with patients positioned in the left lateral decubitus position. For accuracy, all measurements were averaged across three consecutive cardiac cycles, maintaining a frame rate above 50 Hz. Standard parameters evaluating LV morphology and function were assessed according to current clinical guidelines [7,8].

Left atrial (LA) volumes were determined through the biplane Simpson’s method utilizing apical four- and two-chamber views, with volumes indexed to body surface area [9,10]. Specific volumes measured included: (1) LAmax, maximal volume at the end of ventricular systole, just prior to mitral valve opening; (2) LAmin, minimal volume recorded at end-diastole, immediately before mitral valve closure; and (3) LApre-A, volume at the onset of the P-wave on the surface ECG, marking atrial contraction initiation.

To assess LA phasic function, volumetric emptying fractions were calculated: (1) $ \text{Total emptying fraction (LATEF \%)} = \frac{LA_{\text{max}} - LA_{\text{min}}}{LA_{\text{max}}} \times 100 $; (2) $ \text{Passive emptying fraction (LAPEF \%)} = \frac{LA_{\text{max}} - LA_{\text{pre-A}}}{LA_{\text{max}}} \times 100 $; and (3) $ \text{Active emptying fraction (LAAEF \%)} = \frac{LA_{\text{pre-A}} - LA_{\text{min}}}{LA_{\text{pre-A}}} \times 100 $.

The right atrium (RA) was evaluated in parallel, employing the same volumetric and functional analysis protocols [10]. Epicardial adipose tissue (EAT) thickness was quantified by measuring perpendicularly on the free wall of the right ventricle (RV) during end-systole, when maximal wall apposition permits the broadest measurement. Electromechanical delay (EMD) was defined as the interval between the onset of the P-wave in lead II on the ECG, and the peak velocity of the A'-wave on tissue Doppler imaging (TDI) of the lateral LA wall, recorded during sinus rhythm [11].

Speckle-tracking analysis

**Figure 2:**
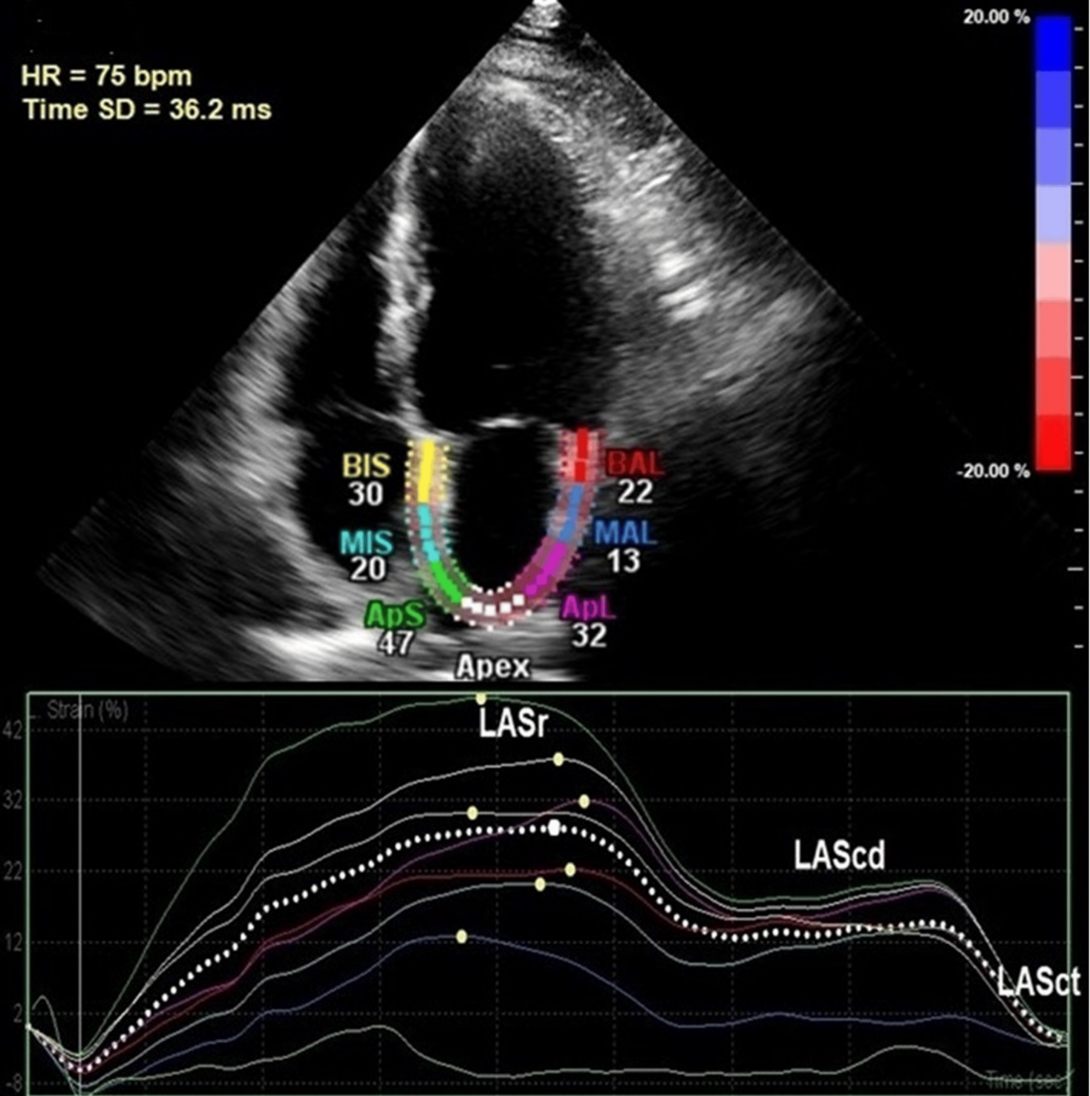
Left atrial strain components in patient with atrial fibrillation LASr, left atrial reservoir strain; LAScd, left atrial conduit strain; LASct, left atrial contractile strain; BAL, basal anterolateral; MAL, mid anterolateral; ApL, apicolateral; BIS, basal interatrial septum; MIS, mid interatrial septum; ApS, apical interatrial septum

Assessment of LA strain was performed using apical four-chamber views, carefully obtained to avoid foreshortening. The strain analysis was conducted offline, utilizing Philips QLAB 10.3 software (Figure 2). LA deformation was segmented into three functional phases [9,10]: (1) reservoir strain (LASr%) - calculated as the strain difference from ventricular end-diastole to the point of mitral valve opening; (2) conduit strain (LAScd%) - defined as the change in strain occurring between mitral valve opening and the onset of atrial contraction; (3) contractile strain (LASct%) - measured as the strain difference between atrial contraction onset and ventricular end-diastole. 

**Figure 3:**
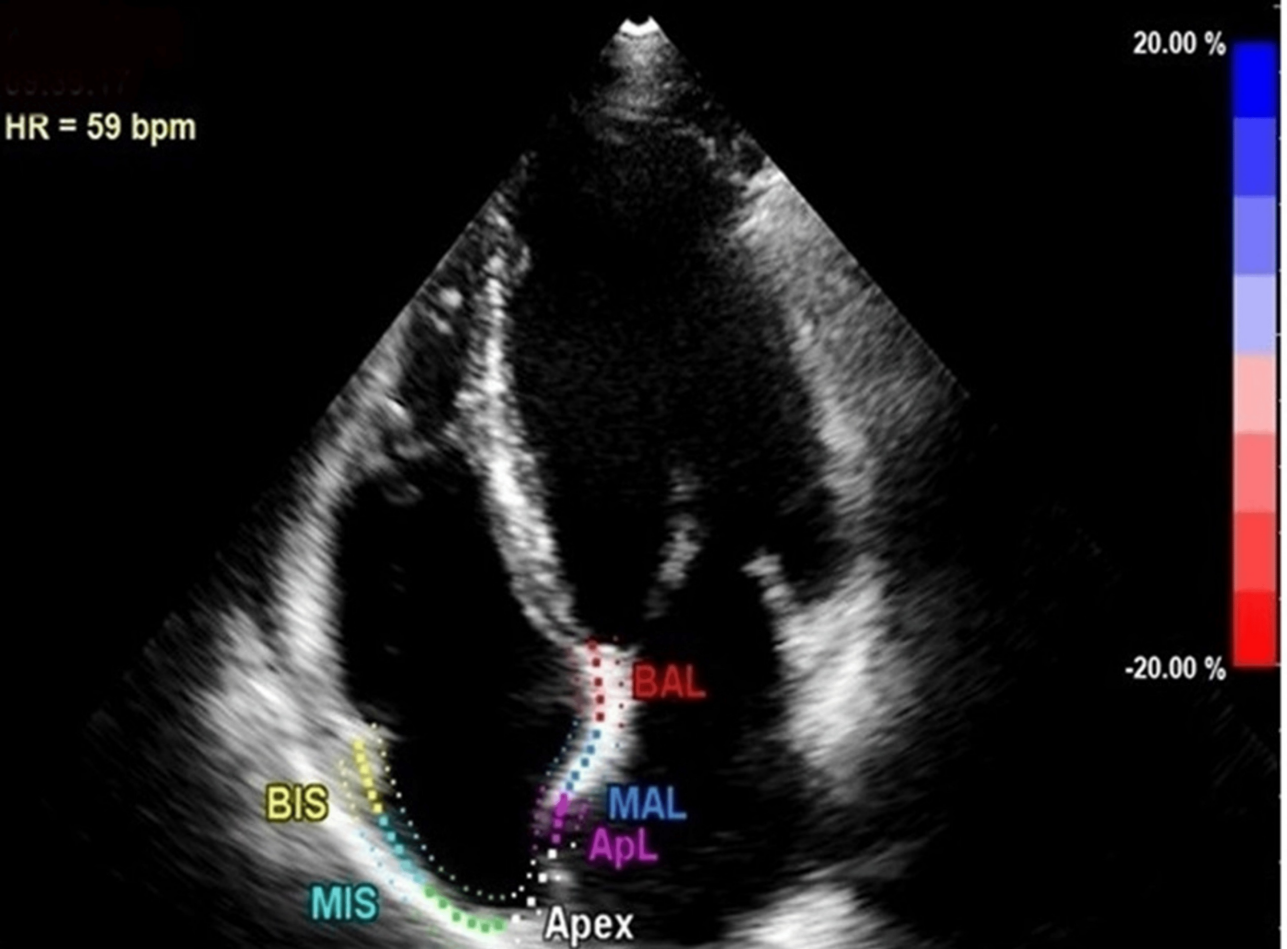
Right atrial strain measurement in patient with atrial fibrillation BAL, basal anterolateral; MAL, mid anterolateral; ApL, apicolateral; BIS, basal interatrial septum; MIS, mid interatrial septum

The stiffness of the LA was quantified by calculating the ratio of the mitral inflow velocity to the mitral annular early diastolic velocity (E/Em), divided by the LASr. RA function was analyzed using an RV-optimized apical four-chamber echocardiographic view (Figure 3). The evaluation of RA strain included two key phases [10]: (1) reservoir phase strain (RASr%) - strain measured during atrial filling; (2) contractile phase strain (RASct%) - strain corresponding to atrial contraction. 

Statistical analysis

Statistical analyses were conducted utilizing IBM SPSS Statistics for Windows, Version 23.0 (released 2015; IBM Corp., Armonk, NY, USA). Quantitative data are expressed as means with corresponding standard deviations, while categorical data are summarized as counts and percentages. Differences in categorical variables were tested using the Chi-square method. For continuous variables, group comparisons were performed via one-way analysis of variance (ANOVA), applying Bonferroni adjustment to control for multiple testing. Correlational analyses employed Pearson’s r for variables following a normal distribution, and Spearman’s rho for non-parametric data. Statistical significance was defined at the alpha level of 0.05, with p-values below 0.001 denoting strong significance.

To identify independent factors linked to AF progression, multivariate linear regression models were developed. Diagnostic accuracy of echocardiographic markers, such as LASr and maximal left atrial volume index (LAVi), was assessed using receiver operating characteristic (ROC) curve analysis, with calculation of the area under the curve (AUC).

Reproducibility and intraobserver consistency were examined by repeating measurements on echocardiographic images from 20 randomly chosen participants. Agreement between repeated measurements was visualized through Bland-Altman plots, and reliability was quantified using intraclass correlation coefficients (ICCs), where values approaching 1 indicate excellent reproducibility.

## Results

The study prospectively included 100 consecutive participants, grouped as follows: 33 individuals diagnosed with new-onset AF (average age, 51.9 ± 8.55 years; 66.7% male), 37 patients presenting with paroxysmal AF (average age, 54.95 ± 6.49 years; 59.5% male), and 30 age-matched healthy controls (average age, 51.43 ± 6.10 years; 50% male). Detailed baseline clinical features for each group are presented in Table 1.

**Table 1:** Baseline clinical caracteristics Data are presented as mean ± standard deviation or number (percentage). Group comparisons for continuous variables were performed using one-way ANOVA (F-statistic reported), while categorical variables were analyzed with the Chi-square test. Statistically significant differences between AF groups and healthy controls are indicated by ^(p < 0.05). AF, atrial fibrillation; ANOVA, analysis of variance

Clinical characteristics	New onset AF (n = 33)	Paroxysmal AF (n = 37)	Healthy controls (n = 30)	p-value	F-value
Age	51.9 ± 8.55	54.95 ± 6.49	51.43 ± 6.10	0.269	8.833
Male, %	66.7^ (22)	59.5^ (22)	50 (15)	0.01	2.384
Hypertension, %	72.7^ (24)	75.7^ (28)	0	0.001	42.136
Obesity, %	100^ (33)	100^ (37)	21.1 (6)	0.001	111.762
Hyperlipidemia, %	15.2^ (5)	32.4^ (12)	6.7 (2)	0.022	4.001
Smoking, %	36.4 (12)	29.7 (11)	26.7 (8)	0.692	0.359
Heart rate, beats/min	71.09 ± 9.44	68.08 ± 7.92^	74.13 ± 8.79	0.021	3.872
Systolic blood pressure, mmHg	129.75 ± 6.44^	129.62 ± 6.83^	118.73 ± 10.28	0.001	13.587
Diastolic blood pressure, mmHg	80.57 ± 5.01^	79.72 ± 5.66^	74.73 ± 6.94	0.001	12.364

Although no overt heart disease was present, significant differences in baseline cardiovascular risk factors were identified among the groups. Notably, hypertension was newly diagnosed during hospitalization in the AF patients, while obesity was already established at the time of study inclusion. Smoking status showed no significant variation between groups. Patients with AF also demonstrated lower heart rates and higher systolic and diastolic blood pressures compared to healthy controls. All AF patients received anticoagulant therapy for at least one month and were on β-blockers, though only 19% were treated with antiarrhythmic medications.

Galectin-3 levels were significantly elevated solely in the paroxysmal AF group, reflecting ongoing atrial fibrosis. Baseline hsTnI concentrations were also higher in paroxysmal AF patients than in those with new-onset AF, indicating myocardial injury and atrial-ventricular stress. No differences were observed in hsCRP levels or LV structural and functional echocardiographic parameters among the groups. Both AF groups exhibited prolonged EMD, consistent with atrial electrical remodeling. EAT thickness was greater in AF patients relative to healthy controls (Table 2).

**Table 2:** Echocardiographic parameters and biomarkers Values are mean ± SD. ANOVA was used for group comparisons. *p < 0.05 vs. new-onset AF; ^p < 0.05 vs. healthy. F-statistic reported. hsTnI, high-sensitivity troponin I; EMD, electromechanical delay; LA, left atrial; LAVi, left atrial volume index; LA min, left atrial minimal volume; LA pre-A, left atrial pre-atrial volume; LASr, left atrial reservoir strain; LASct, left atrial contractile strain; RA, right atrial; RAVi, right atrial volume index; RA min, right atrial minimal volume; RA pre-A, right atrial pre-atrial volume; RASr, right atrial reservoir strain; RASct, right atrial contractile strain; ANOVA, analysis of variance

Parameter	New onset of AF (n = 33)	Paroxysmal AF (n = 37)	Healthy controls (n = 30)	p-value	F-value
Galectin-3, ng/mL	13.67 ± 4.79	15.26 ± 4.71^	11.20 ± 3.52	0.001	7.045
hsTnI, pg/mL	1.42 ± 0.88^	2.48 ± 1.00^*	0.61 ± 0.51	0.0001	17.48
hsCRP, mg/L	1.6 ± 0.16	2.1 ± 0.16	1.6 ± 0.18	0.339	0.339
EMD, ms	49.03 ± 20.69^	47.35 ± 21.91^	34.63 ± 21.37	0.017	4.483
EAT, mm	5.98 ± 1.9^	5.91 ± 1.77^	3.43 ± 0.68	0.001	26.627
LAVi, mL/m^2^	29.88 ± 10.56^	33.16 ± 13.89^	21.02 ± 6.73	0.001	10.325
LA min, mL/m^2^	11.37 ± 6.76^	14.38 ± 8.72^	5.78 ± 2.81	0.001	13.584
LA pre-A, mL/m^2^	19.54 ± 8.17^	21.95 ± 10.12^	11.26 ± 3.13	0.001	16.023
LASr, %	35.34 ± 7.83^	30.38 ± 7.59^*	44.12 ± 8.33	0.001	36.123
LASct, %	-14.02 ± 6.29^	-14.40 ± 6.74^	-18.81 ± 7.10	0.009	4.949
LA stiffness index	0.29 ± 0.11^	0.36 ± 0.16^	0.15 ± 0.02	0.001	25.076
RAVi, mL/m^2^	24.47 ± 8.92^	22.55 ± 7.79^	21.02 ± 7.07	0.001	9.909
RA min, mL/m^2^	11.54 ± 6.28^	10.17 ± 5.32^	7.34 ± 3.28	0.006	5.373
RA pre-A, mL/m^2^	16.56 ± 7.01^	15.42 ± 5.64^	10.64 ± 5.18	0.001	8.544
RA stiffness index	0.16 ± 0.07^	0.15 ± 0.08^	0.10 ± 0.08	0.014	4.471
RASr, %	31.29 ± 9.75^	28.48 ± 9.34^	37.58 ± 10.70	0.001	7.194
RASct, %	-12.80 ± 5.31^	-11.84 ± 5.59^	-16.33 ± 7.67	0.012	4.644

Structural and functional parameters of both the LA and RA, assessed using volumetric analysis, showed significant differences between the two AF groups and healthy controls. Notably, patients with paroxysmal AF demonstrated reduced LASr compared to those with new-onset AF, indicating more advanced atrial remodeling in the paroxysmal AF group (Table 2).

**Figure 4:**
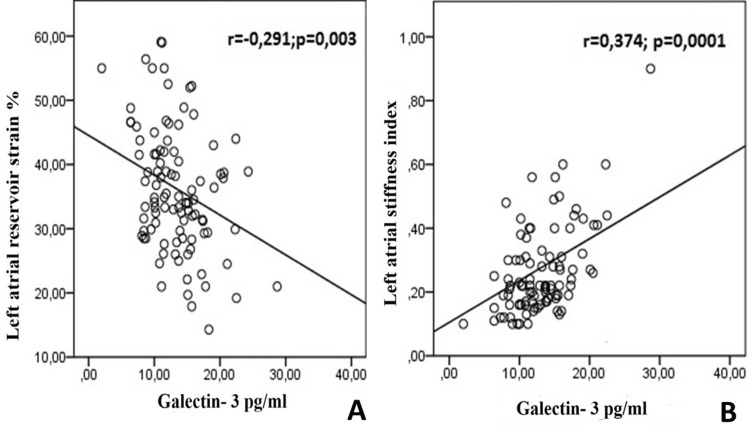
Correlation analysis between galectin-3 (A) Left atrial reservoir strain and (B) Left atrial stiffness index

Significant correlations were observed between galectin-3 levels and LAVi (r = 0.316; p = 0.001), LASr (r = -0.349; p = 0.001), and LA stiffness index (r = 0.374; p = 0.0001) (Figures 4A-4B). 

Multivariable linear regression analysis, incorporating clinical, laboratory, and echocardiographic variables, identified LASr (B = -0.055, p < 0.001), LAVi (B = 0.012, p = 0.05), and LASct (B = 0.041, p = 0.001) as independent predictors of AF progression. Together, these factors explained approximately 43% of the variance in the model (adjusted R² = 0.43).

**Figure 5:**
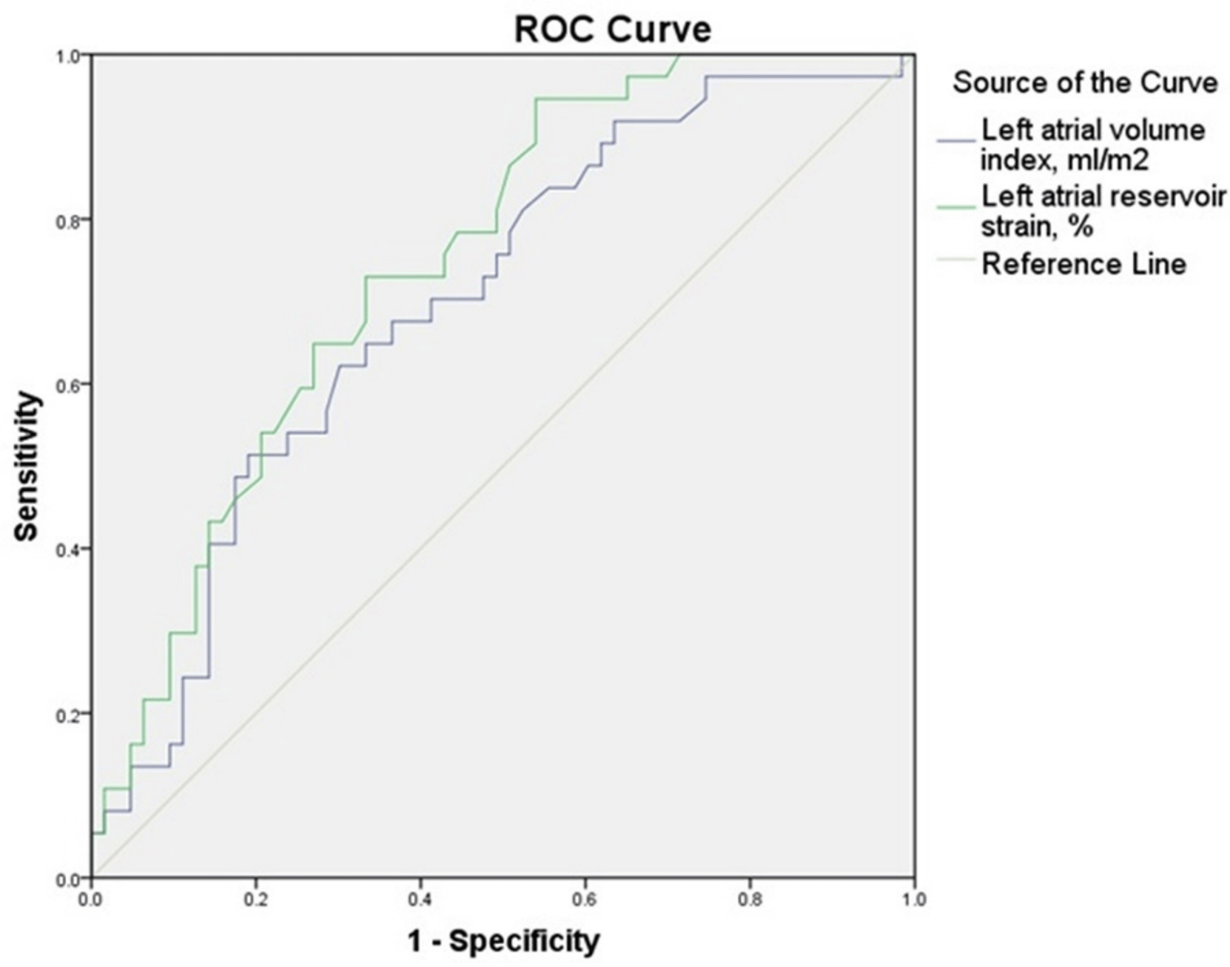
Receiver operating characteristic (ROC) curves for left atrial reservoir strain and left atrial volume index as predictors of atrial fibrillation progression The area under the curve (AUC) was 0.749 (95% CI: 0.654-0.843, p < 0.001) for LASr, and 0.698 (95% CI: 0.593-0.803, p = 0.001) for LAVi, indicating good and moderate discriminatory power, respectively. The ROC analysis demonstrates the utility of LASr and LAVi in identifying patients at risk for AF progression. LASr, left atrial reservoir strain; LAVi, left atrial volume index; AF, atrial fibrillation

ROC curve analysis assessed the predictive accuracy of these parameters. Significant discrimination was observed for LAVi and LASr, with AUC values of 0.698 and 0.749, respectively. In contrast, LASct did not demonstrate significant discriminatory ability (AUC = 0.575, p = 0.213). Therefore, ROC curves for LAVi and LASr are presented in Figure 5. 

Repeatability and reproducibility

To assess intraobserver variability and reproducibility, a second measurement of the same echocardiographic loops was performed after a time interval in 20 randomly selected subjects. Bland-Altman analysis was applied to evaluate agreement, calculating the mean difference (bias) between measurements and the 95% limits of agreement (±1.96 standard deviations). Minimal differences were observed across all LASr and LASct measurements, with the majority falling within the limits of agreement, indicating good repeatability and reproducibility. Additionally, high ICCs - 0.996 for LASr (p < 0.001, 95% CI: 0.989-0.998) and 0.958 for LASct (p < 0.001, 95% CI: 0.889-0.984) - confirmed strong agreement between measurements.

Outcomes

Fifty patients with AF from the initial study cohort were followed for five years to evaluate their clinical progress. At the end of the follow-up, 10% of patients had maintained stable sinus rhythm without AF recurrence. Another 20% underwent pulmonary vein isolation (PVI), although two of these procedures were unsuccessful. Five patients (10%) progressed to permanent AF, indicating advanced atrial electrical and structural remodeling. The majority of patients (60%) experienced frequent AF recurrences, suggesting ongoing or worsening atrial remodeling, and a potentially insufficient response to standard treatment.

## Discussion

Key observations from the present study include: (1) advanced echocardiographic techniques, including volumetric and strain analyses, identified subtle but significant changes in atrial structure and function, predominantly within the LA, suggesting early atrial remodeling in patients with AF and AtCM; (2) among echocardiographic variables, LASr and LAVi emerged as significant independent predictors for the progression of AF. Notably, reservoir strain demonstrated considerable accuracy in differentiating patients at risk; (3) elevated circulating levels of galectin-3 and hsTnI in patients with paroxysmal AF point to increased myocardial fibrosis and injury compared to those with new-onset AF, reflecting a more advanced disease state; (4) long-term follow-up over five years revealed a predominant pattern of recurrent arrhythmia episodes, with a subset of patients advancing to persistent AF, underscoring the progressive nature of atrial remodeling and electrical instability; and (5) the combination of detailed echocardiographic assessment with biomarker evaluation provides a comprehensive framework for early identification and individualized management of AF and related cardiomyopathies in susceptible populations.

AtCM is increasingly recognized as a clinically significant condition due to its potential to contribute to atrial thrombosis, arrhythmias, functional regurgitation, and atrial failure [2-5]. AtCM is generally classified as a staged disease, with expert consensus proposing a classification system for its stages [2]. However, this classification system still requires further validation through clinical studies, and substantial gaps remain in understanding various aspects of the disease process.

Of particular challenge is the identification and differentiation of distinct AtCM phenotypes, primarily due to the limited availability of data and the complex, multifactorial nature of the condition [4]. This limitation complicates efforts to tailor diagnosis and treatment, as different phenotypes may involve distinct underlying mechanisms and clinical manifestations.

Several key stressors contribute to the development of AtCM, with obesity and hypertension emerging as major determinants [1,2,12-14]. These conditions promote distinct pathophysiological mechanisms, including a hemodynamic model driven by hypertension and obesity, as well as metabolic remodeling of the atria, often accompanied by the expansion of EAT [14,15]. Dysfunctional EAT secretes cytokines via paracrine and vasocrine signalling pathways, triggering inflammation, oxidative stress, fibrosis, and electrical remodeling. Collectively, these processes contribute to the initiation and persistence of AF [15].

In our study, obesity and hypertension emerged as primary risk factors for AtCM in middle-aged patients without overt heart disease. Notably, the hypertension observed was newly diagnosed, yet generally mild and well controlled. These conditions induce hemodynamic and metabolic changes in the atria, promoting remodeling processes such as fibrosis, inflammation, and electrical disturbances that drive AtCM progression and increase the risk of AF [14]. Additionally, we observed thicker EAT in both AF groups, further supporting its contributory role in AtCM development.

Previous studies have demonstrated that atrial remodeling, both morphologic and functional, progresses gradually over time [6]. Initially, these changes may remain subclinical, but they are now recognized as part of AtCM Stage 1 [2]. AF may represent the first clinical manifestation of underlying AtCM, and as the disease advances, it can evolve into Stage 2. The onset of AF introduces additional cardiovascular stress, which may ultimately lead to atrial failure [3,5].

Our study included patients with both new-onset and paroxysmal AF, with both groups exhibiting significant alterations in atrial volumes and functional indices compared to healthy controls. These findings suggest that AF may serve as an initial clinical indicator of previously subclinical atrial remodeling. According to the current classification system, both patient groups could be classified as AtCM Stage 2.

LA strain and stiffness are key echocardiographic markers that offer important insights into the mechanical function and structural changes of the LA in patients with AF and AtCM [4,9,15-17]. Both parameters are recognized as significant predictors of disease progression, including AF onset, persistence, and the development of AtCM [9,18,19].

Reduced LA strain is a hallmark of atrial dysfunction and is closely associated with both AF and AtCM [20]. Specifically, decreased strain during the reservoir and contractile phases reflects atrial remodeling processes such as fibrosis, dilation, and electrical alterations, all of which predispose patients to AF. Impaired strain contributes to the electrical and structural remodeling that promotes AF [9,16-19]. As the LA enlarges and its contractility declines, the likelihood of AF increases. Notably, strain reduction serves as an early marker of AtCM, often detectable before the clinical manifestation of AF, and is linked to fibrotic remodeling and mechanical dysfunction, which may lead to atrial failure, thrombogenesis, and other complications [3,9,16].

In our study, LA strain indices, together with LA stiffness, were significantly impaired in both AF groups, indicating the presence of structural and functional atrial remodeling. LA stiffness reflects the atrium’s resistance to deformation and correlates with the degree of fibrosis or rigidity within the atrial wall [18]. As stiffness increases, the LA’s ability to contract and relax diminishes, contributing to diastolic dysfunction and the initiation of AF. Elevated stiffness is associated with impaired atrial compliance, abnormal electrical conduction, and a heightened risk of arrhythmias. This increased stiffness, a key characteristic of AtCM, results from fibrous tissue replacing normal myocardium, which leads to atrial dilation and contractile dysfunction, thereby predisposing patients to AF and thromboembolic events [3,16].

To validate the diagnostic value of these echocardiographic parameters, we conducted ROC curve analysis. Among the variables tested, LASr had the strongest predictive accuracy for AF. This supports its role as an early marker of atrial dysfunction and fibrotic remodeling. LAVi also demonstrated moderate predictive ability, aligning with its role as a volumetric indicator of structural change. In contrast, LASct, though significant in multivariable models, showed limited standalone predictive power, highlighting the importance of combined parameter interpretation for clinical decision-making. These results reinforce the value of LASr and LAVi as accessible, non-invasive tools for identifying subclinical AtCM and predicting AF progression.

The susceptibility of the LA to AtCM is influenced not only by its hemodynamic load and electrical role, but also by complex interactions involving myocardial tissue characteristics and neurohormonal factors [15,19,21,22]. Recent advances in imaging allow detailed evaluation of RA function, revealing its significant contribution to overall atrial performance and arrhythmia risk. Speckle-tracking echocardiography-derived RA strain metrics provide incremental prognostic information beyond conventional measures, particularly in forecasting AF recurrence after therapeutic interventions such as catheter ablation and cardioversion [23,24].

Our findings highlight that compromised RA mechanical function, indicated by reduced strain and increased stiffness, correlates with a higher likelihood of AF persistence and recurrence, emphasizing the need for comprehensive atrial assessment in clinical practice. Furthermore, abnormalities in electromechanical coupling, assessed by TDI-derived EMD, serve as early functional markers of atrial remodeling and dysfunction. Increased EMD reflects a loss of synchrony between atrial electrical activation and mechanical contraction, which disrupts effective atrial performance and facilitates arrhythmogenesis [4,11,25]. This is particularly evident in the early stages of AtCM, where electrical and mechanical impairments precede overt structural changes.

In our study cohort, elevated EMD was a sensitive indicator of subclinical atrial remodeling and predicted progression to persistent AF, even in the absence of significant atrial enlargement. This suggests that EMD monitoring may identify patients at risk for disease advancement, allowing timely therapeutic interventions to mitigate atrial dysfunction and its complications.

Biomarkers such as galectin-3 and hsTnI further enrich this risk stratification paradigm by reflecting ongoing fibrotic activity and myocardial injury within the atria [26-30]. The elevated levels of these markers in patients with paroxysmal AF point to early fibrotic remodeling processes, reinforcing the concept of AtCM as a progressive disease with identifiable early phases [2]. The integration of advanced imaging parameters and circulating biomarkers thus provides a powerful framework for early diagnosis, prognostication, and personalized management of AF and AtCM, paving the way for improved clinical outcomes through targeted interventions.

The clinical follow-up of our AF cohort over five years revealed a clear process of progressive atrial remodeling. This ongoing structural and functional deterioration aligns well with the staged progression of AtCM, as currently understood. Among the 50 patients monitored, only 10% maintained stable sinus rhythm without AF recurrence, while 20% required PVI, with variable success reflecting the complex and evolving atrial substrate. Notably, 10% progressed to permanent AF, underscoring that remodeling and disease progression continue despite early intervention. The majority (60%) experienced frequent AF recurrences, further illustrating the dynamic nature of AtCM and the importance of recognizing this as a continuous process of atrial deterioration across its stages.

Study limitations

We acknowledge that the sample size in our study was relatively limited, primarily due to the strict inclusion criteria focused on middle-aged individuals without previously known overt structural heart disease - historically referred to as patients with "lone AF." While this selective approach enabled a detailed evaluation of early, subclinical atrial remodeling, it inevitably limits the statistical power and generalizability of our findings. The inclusion of both new-onset and paroxysmal AF cases introduced a degree of heterogeneity; however, it also allowed us to explore different stages of atrial remodeling and assess potential trajectories of AF progression. A key strength of the study lies in the extended five-year follow-up, which provided valuable longitudinal data on arrhythmia recurrence, progression to permanent AF, and therapeutic response. Nonetheless, long-term outcome data were available for only a subset of patients, which may limit the robustness of the prognostic analyses. To address these limitations, future prospective studies with larger, more diverse populations, and complete follow-up data are warranted. Such studies are essential to validate the diagnostic and prognostic value of advanced echocardiographic parameters and circulating biomarkers, and to further elucidate the molecular mechanisms underpinning AtCM and the natural history of AF.

## Conclusions

This study underscores the diagnostic potential of combining advanced echocardiographic assessment with biomarkers such as galectin-3 and hsTnI for the early detection of atrial remodeling in AF and AtCM. This integrated approach allows for the identification of subclinical structural and functional changes, particularly in the LA, at a stage when intervention may be most effective. Our findings support a more comprehensive strategy for risk stratification and early treatment, which could ultimately improve long-term outcomes. Given the current absence of validated criteria for staging AtCM, this multimodal approach may serve as a foundation for future classification frameworks and hypothesis generation. Larger, prospective studies are needed to confirm these results and establish standardized protocols for early diagnosis and personalized management of patients with AF and underlying atrial remodeling.
